# Construction of a recombinant eukaryotic expression vector containing PHD3 gene and its expression in HepG2 cells

**DOI:** 10.1186/1756-9966-31-64

**Published:** 2012-08-17

**Authors:** Qi-Lian Liang, Zhou-Yu Li, Yuan Zhou, Qiu-Long Liu, Wen-Ting Ou, Zhi-Gang Huang

**Affiliations:** 1 Department of Oncology, Affiliated Hospital of Guangdong Medical College, Zhanjiang, 524001, China; 2 Department of Radiotherapy, Affiliated Tumor Hospital of Guangzhou Medical College, Guangzhou, 510095, China; 3 Department of Epidemiology, School of Public Health, Guangdong Medical College, Dongguan, 523808, China

**Keywords:** Prolyl hydroxylase domain 3 (PHD3), Hepatocellular cancer (HCC), Hypoxia inducible factor (HIF), Caspase-3

## Abstract

Prolyl hydroxylase domain 3 (PHD3) is a hypoxia inducible factor-α (HIFα) regulator; it degrades HIFα in the presence of oxygen. Recently, there have been an increasing number of studies about the role of PHD3 in proliferation and apoptosis of cancer cells. However, most of the evidence for the role of PHD3 is observational, and little is known of the molecular mechanism. In our current study, we constructed a recombinant eukaryotic expression vector containing the PHD3 gene and detected its biological activity in human hepatoma cell line (HepG2 cells). We successfully constructed a recombinant pcDNA 3.1(+)-PHD3 plasmid; the results showed that PHD3 overexpression could inhibit the proliferation of HepG2 cells and induce apoptosis by activating caspase-3 activity. Our study has provided preliminary materials and data for further investigation of the effect of PHD3 on HepG2 cells.

## Introduction

There are three prolyl hydroxylase domain proteins (PHDs), PHD1, PHD2 and PHD3, that are the key regulators of degradation of hypoxia inducible factor (HIF) in mammals. They are known as HIF-prolyl hydroxylase (HPHs) in *Drosophila* and egg-laying nine (EGLN or EGL-9) in *C. elegans*[1,2]. PHD1 and PHD2 mRNAs are highly expressed in placenta, and PHD3 mRNA is highly expressed in both placenta and heart [3]. In the presence of oxygen, two of the proline residues of HIFα are hydroxylated by PHDs, which allows specific recognition and binding of von Hippel-Lindau tumor suppressor protein (pVHL) and then leads to the subsequent ubiquitination and proteosomal degradation of HIFα [4]. In addition, PHDs play a novel role in tumor progression and development [5], especially PHD3. Recently, an increasing number of studies have indicated that PHD3 is involved in the development and prognosis of cancer [6-10] and also appears to induce apoptosis in cancer cells [11-13]. However, most of these studies are observational, and knowledge of PHD3’s molecular mechanism is still limited. In our current study, we constructed a eukaryotic expression vector containing the PHD3 gene and detected its expression in human hepatoma cell line (HepG2) cells to establish a foundation for future studies.

## Materials and methods

### Materials

Plasmid pcDNA 3.1(+) was obtained from the Central Laboratory of Affiliated Hospital of Guangdong Medical College (Guangdong, China). *E. coli* DH5α was gained from the Pathogenic Biology Laboratory of Guangdong Medical College. Human hepatoma cells (HepG2) were obtained from the Laboratory of Hepatobiliary Surgery. Placenta tissue and the written informed consent for this tissue were obtained from the Operating Room of Affiliated Hospital of Guangdong Medical College. RNAiso Plus, High Fidelity Prime Script™ RT-PCR Kit, TaKaRa Agarose Gel DNA Purification Kit Ver.2.0, DL10,000 DNA Marker, DNA A-Tailing Kit, pMD19-T Simple Vector, DNA Ligation Kit Ver.2.0, Hind III, Xho I, TaKaRa MiniBEST Plasmid Purification Kit Ver.2.0 and SYBR® Prime Script® RT-PCR Kit II (Perfect Real Time) were purchased from TAKARA (Japan). Neonatal Bovine Serum was acquired from Hangzhou Sijiqing Biological Engineering Materials Co., Ltd (China). Dulbecco's modified Eagle’s medium(DMEM)was purchased from Hyclone Company (USA). Lipofectamine™ 2000 was purchased from Invitrogen Biotechnology (USA). DMSO was purchased from Sigma (USA). 3-(4,5-Dimethyl-2-Thiazolyl)-2,5-Diphenyl Tetrazolium Bromide (MTT) was purchased from Sangon Biotech (Shanghai) Co., Ltd (China). Primary rabbit polyclonal anti-EGLN3 antibody was purchased from Jiamay Biotech Company (China). Primary rabbit polyclonal anti-Caspase-3 antibody was purchased from Zhongshan Goldenbridge Biotechnology CO., LTD (China). Primary rabbit polyclonal anti-tubulin antibody, a BCA protein assay kit and BeyoECL Plus were purchased from Beyotime Institute of Biotechnology (China).

### Vector construction

#### Total RNA extraction and PHD3 cDNA synthesis

Total RNA from placental tissue was extracted with RNAiso Plus according to the manufacturer’s instructions. First, 1 μg of total RNA was used to synthesize full-length PHD3 CDS with High Fidelity Prime Script™ RT-PCR Kit. A pair of specific primers, containing Hind III and Xho I restriction enzyme cutting sites, were designed: forward 5^′^-CCCAAGCTTGATGCCCCTGGGACACATCAT-3^′^ and reverse 5′-CCGCTCGAGTCAGTCTTCAGTGAGGGCAGA-3^′^.

#### Purification of PHD3 cDNA and ligation with pMD19-T simple vector

The RT-PCR products were separated with 1.5% agarose gel electrophoresis, and the target fragments were retrieved and purified by TaKaRa Agarose Gel DNA Purification Kit v.2.0. The target fragments were polyadenylated using DNA A-Tailing Kit; these fragments were then ligated into pMD19-T Simple Vector with DNA Ligation Kit v.2.0 (TA Clone). The recombinant pMD19-T-PHD3 was transformed into *E. coli* DH5α competent cells for amplification. Recombinant vectors were isolated from transformants by TaKaRa MiniBEST Plasmid Purification Kit v.2.0, and the pMD19-T-PHD3 was sequenced by an ABI 377 DNA sequencer (Applied Biosystems, USA).

#### Construction of recombinant pcDNA 3.1(+)-PHD3 eukaryotic expression vector

The pMD19-T-PHD3 plasmids were digested by Hind III and Xho I restriction enzymes, and the target fragments (full length PHD3 cDNAs) were isolated and purified. The pcDNA 3.1(+) eukaryotic expression vectors were also digested by Hind III and Xho I and then ligated into PHD3 cDNA with DNA Ligation Kit v.2.0. The recombinant pcDNA 3.1(+)-PHD3 was amplified in *E. coli* DH5α competent cells, and isolated with TaKaRa MiniBEST Plasmid Purification Kit v.2.0. The correct pcDNA 3.1(+)-PHD3 plasmid sequence was verified by restriction enzyme mapping and DNA sequencing.

**Figure 1:**
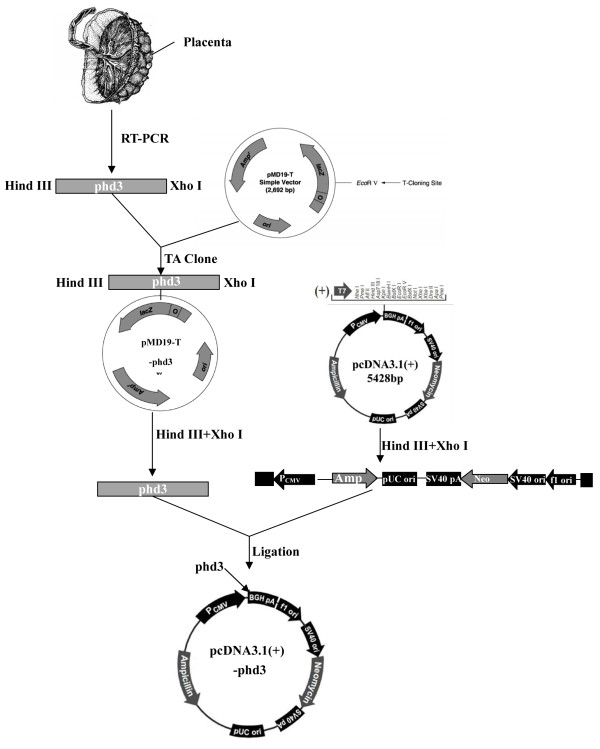
Schematic representation of constructed recombinant pcDNA 3.1(+)-PHD3 eukaryotic expression vector.

A Schematic representation of the construction of the recombinant pcDNA 3.1(+)-PHD3 eukaryotic expression vector is presented in Figure 1.

### Expression of the recombinant pcDNA 3.1(+)-PHD3 eukaryotic expression vector in HepG2 cells

#### Cell transfection

HepG2 cells were cultured in DMEM containing 10% Neonatal Bovine Serum at 37°C in a humidified atmosphere of 5% CO2. Cells were passaged and plated (12-well plates for mRNA assay, 6-well plates for western blot and 96-well plates for growth curve assay) for 24 hours before transfection at 80% –90% confluence. Cells were divided into four groups: no treatment (Normal), Lipofectamine™ 2000 (LP2000), Lipofectamine™ 2000 + pcDNA 3.1(+) (PC3.1) and Lipofectamine™ 2000 + pcDNA 3.1(+)-PHD3 (PHD3). Transfection was carried out according to Lipofectamine™ 2000 instructions. Forty-eight hours after transfection, cells were collected to conduct subsequent assays.

#### Detection of PHD3 mRNA by quantitative real time RT-PCR

Total RNA was isolated from transfected cells by RNAiso Plus, and 500 ng of total RNA was analyzed with SYBR® Prime Script® RT-PCR Kit II on a LightCycler480 (Roche, Switzerland) according to manufacturer’s instructions. The primers were as follows: PHD3 forward 5’- CATCAGCTTCCTCCTGTC-3’, reverse 5’- CCACCATTGCCTTAGACC-3’ and β-actin forward 5’- CTGTGCCCATCTACGAGG-3’, reverse 5’- ATGTCACGCACGATTTCC-3’. The data were analyzed using Ct method.

#### Western blot assay

After transfection, cells were collected and lysed, and the protein concentration was detected by BCA protein assay kit. Supernatants were loaded on a 12%SDS–PAGE gel, and they were then wet transferred onto PVDF membranes. The membranes were incubated with their respective primary antibodies, followed by incubation with HRP-conjugate secondary antibodies. The bands were visualized with BeyoECL Plus and exposed to X-ray film.

#### Cell proliferation assay

To analyze the effects of PHD3 on proliferation of HepG2 cells, MTT assay was performed. Cells were cultured in 96-well plates, and a total cell number was detected every 12 hours. At each time point, twenty μl of MTT (5 mg/ml) was added to each well, and incubated at 37°C for 4 hours. The supernatant was discarded, and 150 μl of DMSO was added to each well. The absorbance (OD value) of the cells was measured using a micro plate reader (Thermo, USA) with a 492 nm filter.

### Statistical analysis

The data were presented as mean ± SD based on three independent experiments. Statistical comparisons between two groups were made by Student’s *t* test, and the cell growth curve was analyzed with multivariate analysis of variance (MANOVA). Statistical analyses were performed by using SPSS 13.0 software for windows (SPSS Inc., USA). Statistical significance was defined as *P* < 0.05.

## Results

### Evaluation of RT-PCR product and recombinant pcDNA 3.1(+)-PHD3 eukaryotic expression vector

**Figure 2:**
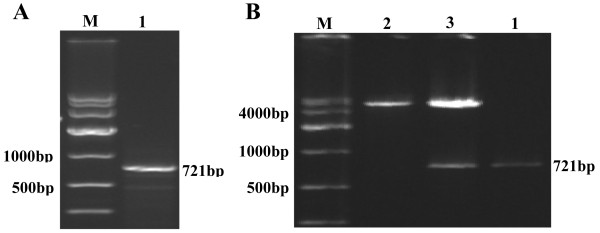
**Identification of PHD3.** (**A**) Electrophoresis of full-length target gene RT-PCR product; M: DNA Marker DL10,000, 1: PHD3. (**B**) Hind III and Xho I digestion and electrophoresis of pcDNA 3.1(+)-PHD3 eukaryotic expression vector; M: DNA Marker DL10,000, 1: PHD3, 2: pcDNA 3.1(+) plasmid digested by Hind III and Xho I, 3: pcDNA 3.1(+)-PHD3 plasmid digested by Hind III and Xho I.

**Figure 3:**
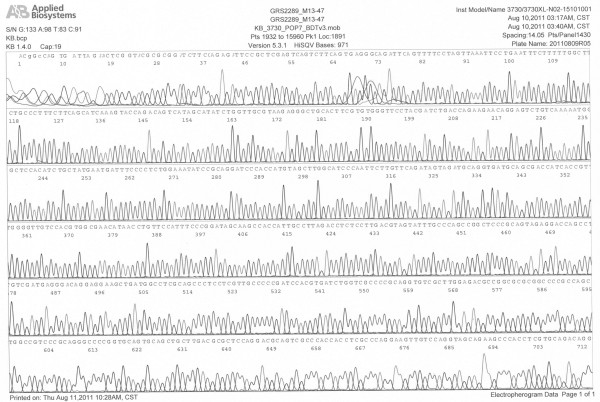
Sequence of full-length 721 bp PHD3 gene.

The RT-PCR products were loaded on 1.5% agarose gels, and the band for full-length PHD3 cDNA was located at 721 bp (Figure 2A). After the PHD3 cDNA fragment was inserted into the pcDNA 3.1(+) plasmid (5428 bp), the fragment was confirmed by Hind III and Xho I digestion and electrophoresis (Figure 2B). Additionally, the cDNA was confirmed by DNA sequencing, as shown in Figure 3.

### mRNA and protein expressions of PHD3 in HepG2 cells

**Figure 4:**
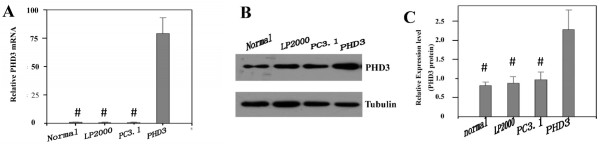
**Expression and biological activity of PHD3. (A) PHD3 mRNA was measured by quantitative real-time RT-PCR.** Cells transfected with PHD3 significantly overexpressed PHD3, compared with the control groups (all *P*=0.00). (**B** and **C**) PHD3 protein was analyzed by western blot. Cells transfected with PHD3 significantly overexpressed PHD3, compared with the control groups (all *P*=0.00). Normal: no treatment, LP2000: Lipofectamine™ 2000, PC3.1: Lipofectamine™ 2000+pcDNA 3.1(+), PHD3: Lipofectamine™ 2000+pcDNA 3.1(+)-PHD3. ^#^*P*<0.05 indicates statistically significant differences in comparison to PHD3-transfected cells.

After transfection, the expression of PHD3 was analyzed by quantitative real-time RT-PCR and western blot. The results showed that the PHD3 transfected group overexpressed more PHD3(all *P* = 0.00), when compared with the control groups (Figure 4A, Figure 4B and Figure 4C).

### Effect of PHD3 on proliferation of HepG2 cells

**Figure 5:**
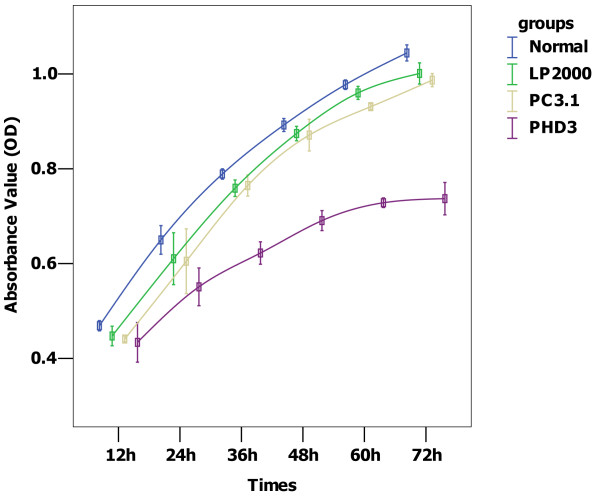
**HepG2 cell growth curves. Compared with the control groups, PHD overexpression significantly inhibited cell proliferation (all*P*=0.00).** Normal: no treatment, LP2000: Lipofectamine™ 2000, PC3.1: Lipofectamine™ 2000+pcDNA 3.1(+), PHD3: Lipofectamine™ 2000+pcDNA 3.1(+)-PHD3.

The OD value of each group was obtained by measuring it every 12 h after transfection, for up to 72 h. Cell proliferation curves were depicted with mean OD values of each time point. As shown in Figure 5, the pcDNA 3.1(+)-PHD3 transfected group grew slower than the control groups (all *P* = 0.00)

### Effect of PHD3 on apoptosis of HepG2 cells

**Figure 6:**
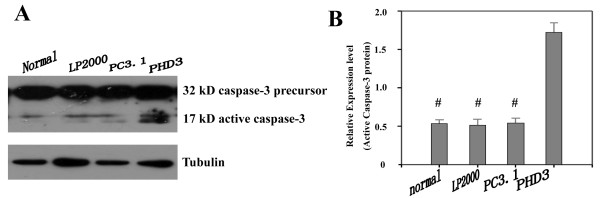
**Activation of caspase-3. Cells transfected with the cleaved 17 kD active caspase-3 fragment of PHD3 expressed more protein than the control groups (all*P*=0.00).** Normal: no treatment, LP2000: Lipofectamine™ 2000, PC3.1: Lipofectamine™ 2000+pcDNA 3.1(+), PHD3: Lipofectamine™ 2000+pcDNA 3.1(+)-PHD3. ^#^*P*<0.05 indicates statistically significant differences in comparison to PHD3-transfected cells.

To investigate whether PHD3 has an effect on inducing apoptosis in HepG2 cells, caspase-3 assays were performed. We found that PHD3 overexpression increased caspase-3 activity (all *P* = 0.00), and the cleaved 17 kD active caspase-3 fragment was visualized by western blot analysis (Figure 6A and Figure 6B).

## Discussion

PHD3 was originally considered an HIFα regulator; it played a vital role in the progression and prognosis of cancer by targeting the degradation of HIFα. Recently, a number of studies have shown that PHD3 was closely related to cancer, independent of its hydroxylase activity. Chen, S et al. [8] found that PHD3 was highly expressed in lung cancer (NSCLC), associating with early-stage and well differentiated tumors. Fox, S. B et al. [14] showed that PHD3 expression was significantly increased after therapy with epirubicin, alone or in combination with tamoxifen, in patients with T2-4 N0-1 breast cancer; however, PHD3 expression was not relevant in treatment response and survival. Su, C et al. [6] also demonstrated that the expression of PHD3 was significantly increased from non-cancerous mucosa to cancer, and its high expression correlated with well differentiated tumors. In contrast, Couvelard, A et al. [10] discovered that high nuclear PHD3 expression related to poor survival in patients with pancreatic endocrine tumors. Gossage, L et al. [9] also found that PHD3 expression in tumor tissue indicated a worse overall disease-free survival in ampullary adenocarcinomas and pancreatic adenocarcinomas. These studies suggested that the role of PHD3 varied from one cancer type to another and that it could be a predictor for treatment and prognosis of cancer. With an increased understanding of PHD3, more attention has been focused on its ability to suppress tumor growth [11-13]; however, little is known about PHD3’s exact mechanism. In pancreatic cells overexpressing PHD3, Su, Y et al. [13] found that apoptosis increased sharply in the presence of nerve growth factor by the activation of caspase-3. Tennant, D. A et al. [12] demonstrated PHD3-mediated alpha-ketoglutarate-induced apoptosis in three human colorectal cancer cell lines (HCT116, A431 and A375). In colorectal cancer cells, PHD3 inhibits cell growth by blocking IKKβ/NF-κ B signaling [11].

So far, the relationship between PHD3 and hepatocellular cancer (HCC) is still unclear. To clarify the effect of PHD3 on HCC, we constructed a recombinant eukaryotic expression vector containing PHD3 and detected its biological activities in HepG2 cells. The results showed that pcDNA3.1(+)-PHD3 was successfully constructed, and PHD3 could be overexpressed in HepG2 cells after transient transfection. To investigate whether PHD3 can inhibit HepG2 cells, we carried out a cell growth curve assay and found that PHD3 arrested cell proliferation. Moreover, we analyzed caspase-3 activity and clarified whether PHD3 had an effect on apoptosis. We found that the cleaved 17 kD active caspase-3 fragment was significantly overexpressed in PHD3 group, which is in line with previous studies [13,15].

In conclusion, we constructed a recombinant eukaryotic expression vector, pcDNA3.1(+)-PHD3, showing that PHD3 overexpression can inhibit proliferation and induce apoptosis in HepG2 cells. Our study has provided preliminary data for further research of stably transfecting pcDNA3.1(+)-PHD3 into HepG2 cell and clarifying the mechanism of PHD3-induced apoptosis.

## Competing interests

The authors declared that they have no competing interest.

## Authors’ contributions

Qi-Lian Liang conceived and designed the study, and drafted the manuscript. Zhou-Yu Li carried out molecular genetic studies and drafted the manuscript. Yuan Zhou Qiu-Long Liu1 and Wen-Ting Ou contributed to cell culture, cell transfection and western blot respectively. Zhi-Gang Huang participated in statistical analyses. All authors read and approved the final manuscript.
